# Concurrence of familial Mediterranean fever and Behçet’s disease: a case report and review of the literature

**DOI:** 10.1186/s13256-023-04185-5

**Published:** 2023-10-22

**Authors:** Adhora Mir, Catherine Ivory, Juthaporn Cowan

**Affiliations:** Department of Medicine, University of Ottawa, 501 Smyth Rd, Ottawa, ON Canada

**Keywords:** Autoinflammatory disease, Behçet’s disease, Case report, Familial Mediterranean fever

## Abstract

**Background:**

Familial Mediterranean fever and Behçet’s disease are distinct disorders that are prevalent in the Mediterranean and Middle Eastern populations. They are characterized by unprovoked inflammatory episodes caused by overexpression of proinflammatory cytokines. Although reported previously, the overlapping presentation of familial Mediterranean fever and Behçet’s disease remains uncommon.

**Case presentation:**

A 46-year-old Lebanese–Canadian man who presented with recurrent oral and genital ulcers, polyarticular synovitis, ocular swelling, recurrent infections, and fevers was later found to have heterozygous mutations of pathogenic *MEFV* c.2080A > G (p. Met 694Val) and c.2082G > A (p.Met694IIe) genes indicating familial Mediterranean fever. He was treated with prednisone, colchicine, and azathioprine, with inadequate symptoms control. Treatment was complicated by recurrent infections.

**Conclusions:**

Our case contributes to the growing literature demonstrating the presentation of predominantly Behçet’s disease-like features in the setting of diagnosis of familial Mediterranean fever. These findings emphasize that clinicians should be aware that patients with familial Mediterranean fever may present with Behçet’s disease-like clinical manifestations.

## Introduction

Familial Mediterranean fever (FMF) is a predominantly autosomal recessive autoinflammatory syndrome that is typically characterized by recurrent fever and serositis [1]. FMF is associated with mutations in the *MEFV* gene, which encodes the protein pyrin. Pyrin is primarily expressed by polymorphonuclear neutrophils and mature monocytes. It assists in regulating inflammation through enhancing interleukin (IL)-1 responsiveness. FMF mutations in *MEFV* result in increased inflammatory flares by increasing the activity of the pyrin inflammasome [2–4]. Common symptoms on presentation include recurrent fevers, acute peritonitis, monoarticular synovitis, rarely pericarditis, and amyloidosis. The five most common mutations include p.Met694Val, p.Met694Ile, p.Val726Ala, p.Met680IleGC, and p.Glu148Gln [5]. There is geographical variability of mutations, for instance, p.Met694Val and p.Met694Ile were found to be the most common mutations in Jordan and Lebanon in one study [6]. The p.Met694Val mutation, particularly when homozygous, has been associated with the development of amyloidosis and more severe disease [7].

Behçet’s disease is a variable-sized vessel vasculitic disease of unknown etiology, characterized by oral and genital aphthae, relapsing uveitis, and various skin eruptions [9]. Similar to FMF, it is predominant in the Middle Eastern and Mediterranean populations. Increased frequency of *MEFV* mutations compared with populations known to be rich in Behçet’s disease has been demonstrated [1], suggesting that MEFV may confer susceptibility to Behçet’s disease. In this paper, we describe a case of FMF presented with features of Behçet’s disease.

## Case report

A 47-year-old, Lebanese–Canadian male with a chronic history of recurrent skin and soft tissue infection requiring intravenous antibiotics, and recurrent pneumonias in the past several months without history of underlying lung disease was referred to the immunodeficiency/infectious disease clinic for immunodeficiency workup. The patient endorsed a remote history concerning systemic immune disease for cyclic fevers with fatigue, chronic otitis media, polyarthritis, recurrent abdominal pain, and aphthous stomatitis (Table 1). These symptoms stopped spontaneously during puberty at approximately age 15 years, at which point they were replaced by a constellation of acneiform rash on his face that recurs annually, daily headaches with inconclusive investigations including negative electroencephalography, and alopecia areata. At age 42 years, his prior symptoms relapsed, with onset of cyclic fevers with fatigue, polyarthritis, aphthous and genital ulcers, and recurrent infections. His infections consisted of pneumonia, chronic otitis media, and mastoiditis. The aphthous stomatitis was initially thought to be caused by dental abscesses; however, they persisted despite total teeth removal. Interestingly, a swab of the ulcer was positive for herpes simplex virus (HSV)-1 deoxyribonucleic acid on one occasion. He also developed intermittent blurry vision of both eyes, assessed by an ophthalmologist who gave a diagnosis of uveitis. At age 47 years, he began to have chest and abdominal pain associated with his intermittent fevers. There was no relevant psychosocial history.

**Table 1:** Timeline of symptomatic presentation

Age 0 to < 15 (years)	Age 15 to < 42 (years)	Age ≥ 42 (years)
• Cyclic fevers with fatigue; average once weekly, self resolving 2–3 days • Otitis media; chronic • Polyarticular arthritis; knee, wrist, ankles • Abdominal pain; inconclusive endoscopy • Aphthous stomatitis	• Alopecia areata • Headaches; daily, inconclusive investigations including electroencephalogram (EEG) • Acneiform rash over face	• Relapse of cyclic fever, fatigue, polyarticular synovitis; every 1–2 weeks • Recurrent ulcers; genital and aphthous (HSV-1 confirmed on one oral swab) • Recurrent infections; pneumonia, otitis media, thrush, skin • Recurrent ocular swelling; onset age 44 years • Splenomegaly; found on CT age 44 years • Ongoing headaches, alopecia areata, and acneiform rash

Splenomegaly was noted on a CT scan of abdomen and pelvis. Laboratory tests revealed maintained antibody titers to measles, mumps, rubella, diphtheria and tetanus; normal levels of immunoglobulin (Ig)G, IgA, and IgM including IgG subclasses; normal T and B cell counts; normal bone marrow (BM) biopsy; and normal complement function, CH50. He had a slightly positive antinuclear antibodies (ANA) (1:80) with no other positive serology. His blood tests also revealed very low vitamin D level and he was treated for microelement deficiencies that may explain his recurrent infections. It was felt that his presentation was in keeping with probable Behçet’s disease, although it was recognized by the clinical team that he did not meet complete diagnostic criteria for Behçet’s disease. HLA-B51 status was not available.

The patient reported that his mother was diagnosed with Behçet’s disease due to chronic mouth sores, upper arm ulcers and abdominal pain. Notably, his sister also endorses chronic ulcers and arthralgias and his 11-year-old son had developed alopecia. He was subsequently referred for gene panel testing for inborn error of immunity due to strong familial phenotypes. Genetic testing revealed heterozygous mutations of pathogenic *MEFV* c.2080A > G (p. Met 694Val) and c.2082G > A (p.Met694IIe) genes. This confirmed the genetic diagnosis of FMF syndrome. Anti-IL-1 blocker was considered as the next therapeutic agent; however, treatment initiation was limited by recurrent infection. He has been restarted on colchicine in the interim. His children were also referred for genetic analysis.

The diagnosis of FMF can be made based on clinical symptoms and supported by genetic testing. There have been several proposed diagnostic criteria [11, 12]; however, the most widely accepted is the Tel-HaShomer criteria, which was further simplified by Livneh *et al*. [13, 14] (Table 2). Our patient was diagnosed with FMF based on the Livneh *et al*. criteria, in that he satisfies at least two minor criteria for incomplete attacks. He also meets five supportive criteria, including appropriate ethnic origin, age < 20 years at disease onset, severe attacks requiring bed rest, spontaneous remission of attacks, and symptom-free interval. His diagnosis is also supported by the presence of pathogenic mutations.

**Table 2:** Diagnostic criteria of familial Mediterranean fever adapted from Livneh *et al*.

*Major criteria*
Typical attacks
(1) Peritonitis (generalized) (2) Pleuritis (unilateral) or pericarditis (3) Monoarthritis (hip, knee, ankle) (4) Fever (rectal temperature 38 °C or higher, lasting between 12 h and 3 days)
*Minor criteria*
(1) Incomplete attacks involving one or more of the following sites: (a) Abdomen (localized, no signs of peritonitis) or (b) chest (2) Joint (arthritis involving other joints) (3) Exertional leg pain (4) Favorable response to colchicine

Supportive criteria include family history of FMF, appropriate ethnic origin, age < 20 years at disease onset, features of attacks including requiring bed rest due to severity, spontaneous remission, symptom-free interval, transient inflammatory response, episodic proteinuria/hematuria, unproductive laparotomy or removal of appendix, consanguinity of parents. Diagnosis with ≥ 1 major criteria, or ≥ 2 minor criteria, or 1 minor criterion plus ≥ 5 supportive criteria, or 1 minor criterion plus ≥ 4 of the first 5 supportive criteria [14].

In accordance with the International Criteria for Behçet Disease (ICBD), the patients symptoms place him in the category of definitive diagnosis of Behçet’s disease with a score of at least four for oral and genital ulcers [15]. Uveitis is also known to be a significant predictor of Behçet’s disease [15]. As such, this patient has both clinical diagnoses of FMF and Behcet’s disease. Notably, body mass index (BMI) > 30 kg/m^2^ has previously been found to be independently associated with incidence of Behcet’s disease [16]. However, the patient’s BMI (21.5 kg/m^2^) was not felt to be contributory. He also did not experience any nonclinical-related challenges during the diagnostic process.

The patient first underwent a trial of colchicine 0.6 mg twice daily for 1.5 months with mild improvement of joint pain only. His fevers were transiently mitigated with high-dose oral prednisone however returned when dose of prednisone was reduced below 20 mg daily. This was discontinued following two subsequent episodes of pneumonia. He was subsequently treated with azathioprine 100 mg daily for 3 months with a decrease of aphthous stomatitis; however, it was ineffective at reducing fevers or joint pain. An anti-IL-1 blocker anakinra was considered as the next therapeutic agent; however, treatment initiation is limited by recurrent infection.

He has been restarted on colchicine 0.6 mg three times daily in the interim, which the patient has yet to begin. At the time of this case report, the patient continues to have acute on chronic otitis media and mastoiditis requiring Port-a-cath for regular intravenous antibiotics. He also continues to have twice weekly fevers, headaches, blurry vision, and joint pain. The patient requires approximately 90 tablets of Tylenol-3 to relieve his pain. There is no current evidence of amyloidosis.

## Discussion

FMF and Behçet’s disease may coexist. Dysregulation of innate inflammatory mechanisms is a hallmark of autoinflammatory conditions, resulting in vasculitic inflammation, recurrent fevers, and increased acute phase reactants. Conversely, autoimmune conditions are associated with the presence of autoantibodies and major histocompatibility complex (MHC) alleles that respond to antigen-specific triggers. While FMF is a prototypal inherited autoinflammatory disease thought to result from inappropriate activation of the proinflammatory cytokine IL-1, the classification of Behçet’s disease into one of these categories is not clear [27, 28]. Although Behçet’s disease has not been found to be associated with autoantibodies, it is related to the HLA-B*51 allele of the class I MHC [29], and infectious triggers have been observed [27]. Furthermore, components of the adaptive immune system, including Th1 and Th17 cells, play an important role in the pathogenesis of Behçet’s disease [28]. Nevertheless, the unprovoked inflammatory episodes caused by overexpression of proinflammatory cytokines in Behçet’s disease are characteristics of autoinflammatory conditions [30]. Similar to FMF, there is increased activity of IL-1 $\beta$ and neutrophils in Behçet’s disease. There is evidence that Behçet’s disease is related to specific autoinflammatory diseases, and particularly FMF [31, 32]. Notably, *MEFV* mutations have been shown to be increased in patients with Behçet’s disease, most frequently p.Met694Val [33, 34].

**Fig. 1:**
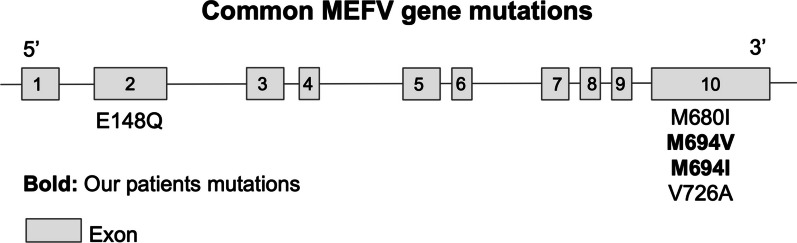
Common *MEFV* gene mutations

Over 70 FMF-associated mutations have been identified in the *MEFV* gene [36]. Of its ten exons, the majority of these mutations are on exon 2 (p.Glu148Gln) and exon 10, with two mutation “hot spots” at codon 680 (p.Met680IleGC) and 694 (p.Met694Val, p.Met694Ile) (Fig. 1). Our patient had a heterozygous genotype of p.Met 694Val and p.Met694IIe. Met694Val is the most common mutation in prevalent populations, accounting for 20–65% of all cases [5]. Mutations at codon 694 and 680, including p.Met694Val, p.Met694Ile, and p.Met680IleGC, have been shown to result in a relatively more severe disease course in patients carrying homozygous genotypes [36]. As this region forms the SPRY domain of pyrin, this supports the hypothesis that this region is critical to the conservation of pyrin structure [5]. Met694Val, especially when homozygous, has been linked with more frequent development of amyloidosis, earlier age of onset, and increased frequency of arthritis [7, 8]. However, some studies have not found these associations [37, 38]. Although our patient did have an early age of onset of disease, he did not have severe features including amyloidosis nor significant arthritis, which are typically associated with these mutations.

Certainly, epigenetic factors such as increased methylation of *MEFV* CpG islands have also been implicated in FMF patients [39]. Modifying loci outside of the *MEFV* gene, including the MHC class I chain-related gene A (MICA) or SAA-1 alpha/alpha alleles, have also been shown to affect age of onset, and risk of developing amyloidosis, respectively, in those with existing *MEFV* mutations [5]. Finally, significantly decreased mRNA expression has been demonstrated in FMF patients, particularly in carriers of the M694V mutation and more severe disease [40]. Altogether, the molecular mechanisms of FMF pathogenesis are only partially elucidated, and pathogenesis may include a complex combination of protein instability, decreased quantity of gene expression, genetic, and epigenetic factors, as well as *MEFV*-independent modifiers.

*MEFV* gene mutations have also been associated with non-FMF inflammatory diseases, including inflammatory bowel disease, rheumatoid arthritis, fibromyalgia syndrome, Behçet’s disease, ulcerative colitis, ankylosing spondylitis, and juvenile idiopathic arthritis [35]. Chiefly, p.Met 694Val, p.Met680IleGC, p.Val726Ala, and inconsistently p.Glu148Gln have been found to occur more often in patients with Behçet’s disease than control populations [1, 41, 42]. Additionally, the silent polymorphism p706 on exon 10 of MEFV was associated with probable Behçet’s disease but not definite Behçet’s disease [1]. The presence of p.Met694Val in our patient correlates with previous literature of coexistence of this mutation in patients with Behçet’s disease. However, p.Met694IIe, though previously reported [41], has not been shown to be significantly elevated in this population.

Although pathogenesis of Behçet’s disease is unclear, PCR and *in situ* hybridization studies have isolated various viruses in patients with Behçet’s disease, including HSV, varicella zoster virus, cytomegalovirus, Epstein–Barr virus, human herpes virus 6 and 7, hepatitis virus, human immunodeficiency virus, and parvovirus [44]. HSV has been detected in peripheral blood leukocytes, saliva, and genital ulcer biopsy [46]. There are also clinical similarities between ulcers caused by HSV and herpetiform ulcers in Behçet’s disease. An animal model observed Behçet’s disease-like symptoms most commonly including skin ulcers, eye symptoms, hair loss, and genital ulcers in up to half of mice with HSV inoculated to earlobes [47]. However, observation from HSV inoculation in this study is insufficient to explain the pathogenesis of Behçet’s disease [47]. Several studies have also implicated HSV in the etiology of Behçet’s disease; however, it remains unclear whether the virus itself or a secondary immunological reaction may contribute to the pathogenesis [48]. Interestingly, HSV was identified from an oral ulcer in our case. HSV might have some implication on our patient’s clinical presentation of Behçet’s disease-like features and with the relapse of cyclic fevers and polyarticular arthritis again at age of 42 years.

To investigate whether FMF and Behcet disease coexist, a literature review was performed. We searched relevant articles using the words “Familial Mediterranean fever and Behçet’s disease; FMF; Familial Mediterranean fever syndrome; Behçet’s disease” from 1980 to 2022 in English language on the PubMed database [17–26].

Nine case reports were identified (Table 3). Only two reported cases did not present with abdominal pain; however, it is unclear whether there was a relevant remote history in these cases [24, 25]. The timing of symptoms differ from our patient, chiefly in the remission of several symptoms following puberty and subsequent relapse in later age. No specific bacteria was identified in the onset of his symptoms, although HSV-1 was confirmed from an oral ulcer. Only one report commented on concomitant occurrence of hydatid disease during symptom flare [23]. No reports thus far have implicated a specific organism in the pathogenesis of triggering Behçet’s disease in patients with FMF. When genetic testing was completed, reported *MEFV* mutations were discrepant and included Met693Lys, Met694Val, and Glu148Gln, as well as the compound heterozygous mutations Met694Val/Arg202Gln, Glu148Gln/Pro369Ser, and Glu148Gln/Met694Val. Only one patient was HLA-B51 positive [21]. In all cases, colchicine was trialed, and was a component of improved symptoms in the majority of reported patients. Colchicine was used in combination with a variety of anti-inflammatory therapies including steroids, nonsteroidal anti-inflammatory drugs (NSAIDs), sulfasalazine, azathioprine, and methotrexate. In one patient in whom the above combinations were unsuccessful, symptom improvement was achieved with the addition of the IL-1 inhibitor anakinra [25].

**Table 3:** Summary of case reports with overlap of familial Mediterranean fever and Behçet’s disease [17–26]

Citation	Patient	Symptomatology (duration)	Treatments	Outcomes
Zerkaoui *et al*. [19]	10F Origin: Morocco p.M693K	Periodic fever with abdominal pain, pseudo erysipelas, vomitus, monoarthritis, headache, one episode aphthous and urogenital ulcers (months), cerebral venous thrombosis (acute)	Colchicine intravenous Methylprednisolone × 3 days, followed by oral prednisone Enoxaparin	Intolerance to colchicine—response not determined Resolution of thrombus at 6 months Papillary edema with scleromalacia on ophthalmology follow-up
Güler *et al*. [20]	19F Origin: Not stated M694V/R202Q	Recurrent aphthous and genital ulcers, erythema nodosum (> 10 years) Recurrent attacks of fever, abdominal pain, arthralgia (2 years)	Colchicine 1.5 mg/day × 10 years (before admission) Colchicine 2 mg/day + methylprednisolone 16 mg/day, azathioprine 100 mg/day, diclofenac sodium 150 mg/day	Recurrent oral aphthae and genital ulcers despite colchicine 1.5 mg/day At 1 month follow-up on the new regimen, improved mucocutaneous ulcers, resolved acute phase reactants. Methylprednisolone gradually decreased to 8 mg/day. At 6 months follow-up, decreased severity and frequency of attacks
Sunar *et al*. [21]	43 M Origin: Not stated HLA-B51 positive	Aphthous stomatitis, inflammatory low back pain, arthralgias, febrile attacks with abdominal pain, genital ulcerations, erythema nodosum, sacroiliitis (> 8 years diagnosed FMF + spondylosing arthritis)	Sulfasalazine 2 g/day, colchicine 4 × 0.5 g and acemetacin 120 mg/day 8 years	Frequency of attacks decreased following colchicine treatment
Mobini *et al*. [22]	27 M Origin: Iran E148Q/P369S	Aphthous and genital ulcers, pustular folliculitis (5 years) Attacks of fever, chills, arthralgia, arthritis (5 months)	Colchicine (5 years) Added prednisolone and azathioprine during admission	Symptoms continued, azathioprine and colchicine discontinued due to new diarrhea and colchicine reintroduced when diarrhea resolved with symptom improvement
Erdem *et al*. [23]	23 M Origin: Not stated	Knee pain, swelling, abdominal pain	Colchicine	Improved knee pain and swelling, no abdominal pain with colchicine; concomitant occurrence hydatid disease
Frigui *et al*. [24]	35 M Origin: Tunisia HLA-B51 negative E148Q	Abdominal pain attacks with fever (15 years), hip and back pain (10 years), Recurrent oral ulcers (2 years), genital ulcers (1 year), thrombosis left popliteal vein (6 months)	Colchicine 1 mg/day and sulfasalazine 2 g/day	Diagnosed ankylosing spondylitis Decreased frequency of attacks at 5 year follow-up
Bilginer *et al*. [25]	17F Origin: Not stated M694V	Recurrent oral ulcers, erythema nodosum, fevers, arthritis (11 years) Secondary amyloidosis (acute)	Colchicine, nonsteroidals, methotrexate, occasional corticosteroids × 11 years Anakinra 1 mg/kg/day, colchicine, isoniazid prophylaxis	Minimal symptom improvement on initial treatment × 11 years 6 month and 1 year follow-up of anakinra, colchicine, isoniazid prophylaxis with resolution of clinical symptoms and acute phase reactants. Worsening proteinuria at 18 months
Matsuda *et al*. [26]	25 M Origin: Japan HLA-B51 negative E148Q/M694V	Buccal aphthous, genital ulcers, iridovirus, fever and thoracoabdominal pain due to pleural peritonitis (10 years)	Oral prednisolone, intermittently in past attacks Colchicine 0.5 mg/day	No improvement on prednisolone Symptoms improved on colchicine
Birlik *et al*. [18]	37 M, Origin: Turkey HLAB5 negative	Recurrent abdominal pain/fever (17 years), Recurrent oral ulcers (2 years), recurrent genital ulcers (1 year), erythema nodosum (9 months), sacroiliitis (acute)	Colchicine 1.5 mg/day and Sulfasalazine 2 g/day	Decreased acute phase reactants at 1 week; decreased acute attacks at 1 year

Evidence of a true association between FMF and Behçet’s disease is still lacking. In a retrospective analysis of the medical and genetic records of 2000 patients in Turkey [43], comorbidities in FMF were assessed and categorized as comorbidities directly related to FMF, comorbidities due to increased innate inflammation, and comorbidities that were regarded as being incidental. Within the category of diseases due to increased innate inflammation, the most prevalent comorbid diseases included ankylosing spondylitis (155 patients), juvenile idiopathic arthritis (31 patients), and IgA vasculitis (25 patients). Behçet’s disease was only associated with FMF in 3 of 2000 patients, which is not higher than the prevalence in the population of Turkey. In contrast, an earlier retrospective analysis of 4000 patients with FMF in Israel found that 16 patients also had Behçet’s disease [49]. As these patients had a kin with either Behçet’s disease or FMF, it was suggested that two distinct diseases occurred at once, rather than one disease with additional manifestations. One noted limitation of this study was the predominance of pediatric patients, in whom the prevalence of Behçet’s disease is known to be lower. However, as the prevalence of Behçet’s disease in Israel was not known, an incidental occurrence could not be ruled out. The prevalence of HLA-B*51 in this population was also lower than previously reported prevalence in geographically similar patients with Behçet’s disease. Even so, as the prevalence in this study was greater than those previously reported in countries with a high prevalence of Behçet’s disease, the authors concluded that Behçet’s disease should be included in vasculitides associated with FMF. Furthermore, in a more recent cross-sectional study of 892 adult patients with Behçet’s disease and their age and sex matched controls in Israel, diagnosis of Behçet’s disease was found to be independently associated with FMF on multivariate analysis (OR 25.16, 95% CI 13–53.3) [32]. As well, the association was found to be predominant in females, people of Arab descent, and those with BMI > 30 kg/m^2^.

Other rare diseases should be considered in the differential of Behçet’s disease and FMF. For instance, A20 haploinsufficiency is caused by mutations in the TNFAIP3, and is an auto-inflammatory disease mediated by the NFkB pathway with many clinical similarities to Behçet’s disease; however, it is typically juvenile onset with excess production of proinflammatory cytokines during flares [50, 51]. Unfortunately, whole-genome sequencing is required to identify such variants, which was not available for our patient.

## Conclusions

Several common FMF related *MEFV* mutations have been associated with Behçet’s disease. As well, Behçet’s disease has been observed to occur more frequently in patients with FMF compared with the general population. It is unclear, however, whether this association is attributable to generalized increased innate immune response in FMF or true shared pathogenesis. Although reported previously, the overlapping presentation of FMF and Behçet’s disease remains uncommon. Nine case reports were found on the coexistence of the FMF and Behçet’s disease. Previous literature has implicated HSV in the etiology of Behçet’s disease and HSV was identified in our patient, which differs from reported cases of Behçet’s disease in patients with FMF. While similar patents have shown symptomatic improvement with colchicine and adjunctive anti-inflamatory therapies, this remains to be seen in our patient. Our case contributes to the growing literature demonstrating the presentation of predominantly Behçet’s disease-like features in the setting of diagnosis of FMF. These findings emphasize that clinicians should be aware that patients with FMF may present with Behçet’s disease-like clinical manifestations.

## Data Availability

Not applicable.
